# Mapping and Ablation of Idiopathic Ventricular Fibrillation

**DOI:** 10.3389/fcvm.2018.00123

**Published:** 2018-09-18

**Authors:** Ghassen Cheniti, Konstantinos Vlachos, Marianna Meo, Stephane Puyo, Nathaniel Thompson, Arnaud Denis, Josselin Duchateau, Masateru Takigawa, Claire Martin, Antonio Frontera, Takeshi Kitamura, Anna Lam, Felix Bourier, Nicolas Klotz, Nicolas Derval, Frederic Sacher, Pierre Jais, Remi Dubois, Meleze Hocini, Michel Haissaguerre

**Affiliations:** ^1^Electrophysiology and Ablation Unit, Bordeaux University Hospital (CHU), Pessac, France; ^2^IHU Liryc, Electrophysiology and Heart Modeling Institute, Foundation Bordeaux Université, Bordeaux, France; ^3^Department of Cardiology, Sahloul Hospital, Universite de Sousse, Sousse, Tunisia; ^4^Department of Cardiology, Royal Papworth Hospital NHS Foundation Trust, Cambridge, United Kingdom

**Keywords:** idiopathic ventricular fibrillation, mapping, ablation, Purkinje, localized substrate

## Abstract

Idiopathic ventricular fibrillation (IVF) is the main cause of unexplained sudden cardiac death, particularly in young patients under the age of 35. IVF is a diagnosis of exclusion in patients who have survived a VF episode without any identifiable structural or metabolic causes despite extensive diagnostic testing. Genetic testing allows identification of a likely causative mutation in up to 27% of unexplained sudden deaths in children and young adults. In the majority of cases, VF is triggered by PVCs that originate from the Purkinje network. Ablation of VF triggers in this setting is associated with high rates of acute success and long-term freedom from VF recurrence. Recent studies demonstrate that a significant subset of IVF defined by negative comprehensive investigations, demonstrate in fact subclinical structural alterations. These localized myocardial alterations are identified by high density electrogram mapping, are of small size and are mainly located in the epicardium. As reentrant VF drivers are often colocated with regions of abnormal electrograms, this localized substrate can be shown to be mechanistically linked with VF. Such areas may represent an important target for ablation.

## Introduction

Idiopathic ventricular fibrillation (IVF) is a rare cause of sudden cardiac death (SCD). It is reported in 6.8% of all patients who survive an out-of-hospital cardiac arrest and is more frequent in young adults (1). Indeed up to 35% of cases of sudden death remain unexplained in patients between 18 and 35 years old (2). Current guidelines define IVF as a diagnosis of exclusion in patients who have survived a VF episode without any identifiable structural or metabolic cause (3). An implantable cardioverter defibrillator (ICD) is usually recommended for primary and secondary prevention of SCD in this population (3, 4). However, around one third of patients with IVF, experience VF recurrence in the 5 years following diagnosis (5). VF ablation is recommended to prevent VF recurrence and reduce the number of ICD shocks (3, 4).

We aim to review the mechanisms underlying IVF and the different ablation strategies in this setting.

## VF pathophysiology

### VF initiation

VF is initiated by premature ventricular complexes (PVCs) or by the transition from a ventricular tachycardia (VT). In patients with IVF, PVCs that trigger the arrhythmia originate from the Purkinje system in up to 93% of the cases (6, 7). More rarely, they originate from the ventricular myocardium including the right ventricular outflow tract (RVOT) (7–11) or the papillary muscle (12, 13). These PVCs may result from abnormal automaticity, triggered activities, or more rarely from reentry, either phase 2 reentry (14) or reentry using the Purkinje system (15). Purkinje cells have distinctive anatomical and electrophysiological properties (16). Abnormal automaticity in the Purkinje fibers likely results from a deficient calcium regulation by the sarcoplasmic reticulum (16, 17). Triggered activities such as early afterdepolarizations (EADs) or delayed afterdepolarizations (DADs) are commonly recorded in the Purkinje cells (18–20) and can result from Ca^2+^ overload (17). These arrhythmogenic mechanisms become more prevalent in the presence of electrolyte imbalance, exposure to drugs, and in the presence of myocardial ischemia (21).

### VF maintenance

Mechanisms that maintain VF are as yet, incompletely elucidated. Animal studies suggest reentrant activities and multiple wavelets as main mechanisms maintaining early VF (22) and Purkinje system as principal mechanism that maintains long duration VF (23). Structural heterogeneities are critical for the occurrence of reentries by decreasing the conduction velocities and thereby anchoring reentries (24–26). Complex myocardial fiber arrangement at the papillary muscle insertions and at the Purkinje tissue can maintain fibrillatory activities in the absence of additional pathology (27). In a mammalian 3-dimensional model, Berenfeld et al. (28) simulated the evolution of reentrant activity at the Purkinje-muscle junction and demonstrated that Purkinje activity is essential to the reentry at its initial stage and led to intra-myocardial reentries that sustained the arrhythmia. Subsequently, Pak et al. (29) demonstrated the contribution of both Purkinje activities and the ventricular myocardium (left postero-septum and papillary muscles) in maintaining VF. Newton et al.(30) and Tabereaux et al. (31) demonstrated that Purkinje fibers are highly active during the VF, mainly 1 min after the initiation. This activation was associated with an endocardial to epicardial gradient (32) and is explained by the resistance of the Purkinje cells to prolonged ischemia. Additional evidence supporting the role of Purkinje fibers in the initiation and maintenance of VF comes from canine heart studies in which chemical ablation of the Purkinje fibers using Lugol's solution significantly increased the VF induction thresholds (33) and was associated to early VF termination (34).

### Genetics of IVF

Several familial cases of IVF have been reported, suggesting that a subset of IVF is hereditary and has a genetic transmission. This has been demonstrated by Alders et al. (35) who performed a genome wide haplotype sharing analysis to identify the responsible gene for IVF in 3 distantly related families from the Netherlands. The authors identified a mutation located on the chromosome7q36 harboring a part of the dipeptidyl peptidase-like protein-6 (DPP6) gene that encodes for a component of the transient outward current (36). The correlation between DPP6 mutation and IVF was confirmed in a larger population of 26 families including 601 family members from the Netherlands (37). The mutation increased levels of the DPP6 mRNA 20 fold compared to controls. Xiao et al. (38) demonstrated that DPP6 overexpression selectively increases the I_TO_ current in the Purkinje fibers leading to abnormal depolarization which may explain a part of the pathogenesis of VF in this group. Other genes have been linked to IVF including CALM1 (39), RYR2 (40), IRX3 (41).

Whole exome sequencing represents the latest approach to genetic testing in patients with IVF, allowing diagnosis of a wide range of sudden death-susceptibility genes (42, 43). Of note however, genetic screening frequently reveals rare variants and variants of uncertain significance that require further classification (20, 44–49).

## Mapping and ablation of VF triggers

### Clinical experience

So far, mapping and ablation of the premature ventricular contractions (PVCs) triggering VF remains the gold standard for IVF ablation. Multiple cases of successful ablation of triggering PVCs have been reported and are represented in Table 1. Ashida et al. (50) first reported successful ablation of right ventricular outflow tract (RVOT) triggers in a patient with recurrent VF episodes. PVC morphology was reproduced by pace-mapping at the septal RVOT. Ablation at this site abolished the arrhythmia and the episodes of syncope. Later, Kusano et al. (51) and Takatsuki et al. (52) also reported successful ablation of ectopics triggering VF arising from the RVOT; this was associated with freedom from VF recurrence. Additional sites of PVCs triggering VF have been reported at the infero-lateral RV (53, 54), Purkinje system (55–57, 59–63), moderator band (67), and papillary muscles (12, 13).

**Table 1:** Case reports of successful ablation of PVCs triggering VF.

**References**	**Patient history**	**Mapping and ablation**	**Outcome**
Ashida et al. (50)	18 y.o female Syncope	Septal RVOT	No VF recurrence after 3 years
Kusano et al. (51)	65 y.o female Syncope	RVOT	No VF recurrence after 18 months
Takatsuki et al. (52)	62 y.o male	Postero-septal RVOT	No VF recurrence after 20 months
Saliba et al. (53)	41 y.o female	PVC coupling interval = 240 ms Duration 140 ms Inferolateral border of the right ventricle Late sharp potential recorded in sinus rhythm and preceding the PVCs	No VF recurrence after 6 months
Betts et al. (54)	27 y.o male Coupling interval 260–300 ms	Free wall of the RVOT Sharp potential 80 ms before PVC onset	No VF recurrence after 11 months of follow-up
Pasquie et al. (55)	3 patients, mean age 62 y.o VF during fever episodes	Coupling intervals = 240 and 320 ms Purkinje potential preceding the PVC (anterior RV)	No VF recurrence after 9, 18 and 22 months
Kohsaka et al. (56)	21 y.o female Electrical storm	Purkinje from the right bundle preceding the PVC initiation VF by 72 ms	No VF recurrence after 12 months
Naik et al. (57)	24 y.o male Syncope	Coupling interval = 280–320 ms 2 PVC morphologies = RVOT + RV apex Few PVCs recorded during the procedure Ablation based on pacemapping and targeting Purkinje potential in the RV apex	VF recurrence after 9 months due to PVCs of similar morphology Redo ablation was associated with VF Freedom after 1 year-follow-up
Cho et al. (58)	17 y.o male Aborted sudden cardiac death due to IVF	Coupling interval = 360 ms Ablation at the anterolateral wall of the RVOT based on the earliest activation site and pacemapping	Acute success with no VF/PVT recurrence during the 2 weeks after the procedure
Szumowski et al. (59)	25 y.o female syncope 150 ICD therapies in 9 years	PVCs originating from the Purkinje network	No VF recurrence after 2 years
Saba et al.(60)	10 y.o male syncope Atrial fibrillation 30 ICD shocks in 2 months	4 PVC morphologies: 2 short coupled (268+/110 ms) with a large QRS (161 ± 7 ms) 2 longer coupled PVCs (422 ± 25 ms) and narrower QRS (118 ± 9 ms) Mapping performed using a basket catheter	No VF recurrence after 21 months using quinidine
Santoro et al.(13)	5 patients, mean age 39 ± 12 years Multiple ICD shocks and electrical storm	PVCs arising from the left ventricular posteromedial papillary muscle in 4 cases and from the right ventricular postero lateral papillary muscle in 1 case.	No VF recurrence after 58 ± 11 months
Nagase et al.(61)	29 y.o female Multiple ICD shocks for VF episodes	PVC with different morphologies Ablation targeting earliest anterior and posterior Purkinje potentials	Recurrence of 3 VF episodes after 96 months No VF recurrence after administration of atenolol and disopyramide
Kleissner et al.(62)	Male Electrical storm	2 PVCs initiating VF The first arising from the right Purkinje preceding the PVC by 28 ms. The second arising from the RVOT.	–
Rosu et al., (63)	39 y.o male Multiple syncopes	PVCs arising from the right Purkinje preceding the PVC by 15 ms.	2 early recurrences of VF episodes initiated by PVC s from the RV with different morphologies. No VF recurrence after 3 years
Chan and Sy (64)	2 patients = 40 and 24 y.o females syncope and cardiac arrest respectively	PVCs arising from the posterior fascicle in the first case and from the RVOT in the second case	No VF recurrence after 17 and 42 months respectively
Ho et al. (65)	44 y.o male Electrical storm	PVCs arising from the moderator band mapped using the Pentaray catheter Purkinje potentials preceding the PVC by 103 ms Ablation targeted Purkinje potentials at the moderator band	No VF recurrence after ablation
Martin et al. (66)	32 y.o male Syncope	PVC arising from the posterior fascicle Purkinje potentials preceding the PVC by 34 ms Ablation based on pacemapping and the site of earliest activation	Recurrence of 1 VF episode after 2 year follow-up

*ICD, implantable cardioverter defibrillator; PVC, premature ventricular contraction; PVT, polymorphic ventricular tachycardia; RV, right ventricle; RVOT, right ventricular outflow tract; VF, ventricular fibrillation*.

So far, two large studies of IVF ablation have been published. The first study included 27 patients with recurrent episodes VF (7). A Purkinje origin was demonstrated in 23/27 (93%) patients. This was located in the left ventricular septum in 10 patients, in the anterior right ventricle in 9 patients, and in both ventricles in 4 patients. The second study was a multicenter study that included 38 patients (11). The PVC origin was in the Purkinje system in 33/38 (87%) patients. They arose from the left Purkinje fibers in 14 patients, from the right Purkinje fibers in 16 patients and from both chambers in 3 patients. A myocardial origin was identified in 5 patients, the majority being from the RVOT (4/5).

In other studies, Noda et al. (10) explored 101 patients with normal structural hearts who presented with PVCs arising from the RVOT. Among this group, 16 patients presented with spontaneous episodes of VF (5 cases) and syncope (11 cases). Ablation targeting the PVCs from the RVOT eliminated episodes of syncope and VF in all patients during a follow-up period of 54 ± 39 months. Santoro et al. (13) explored 5 patients with IVF using intracardiac echocardiography (ICE). They identified a PVC origin in the left ventricular posteromedial papillary muscle in 4 cases and in the right ventricular posterolateral papillary muscle in 1 case. Sadek et al. (67) mapped PVCs triggering VF in a group of 36 patients with VF using ICE. They identified the PVC origin at the moderator band in 7 patients.

### Mapping of VF triggers

**Figure 1:**
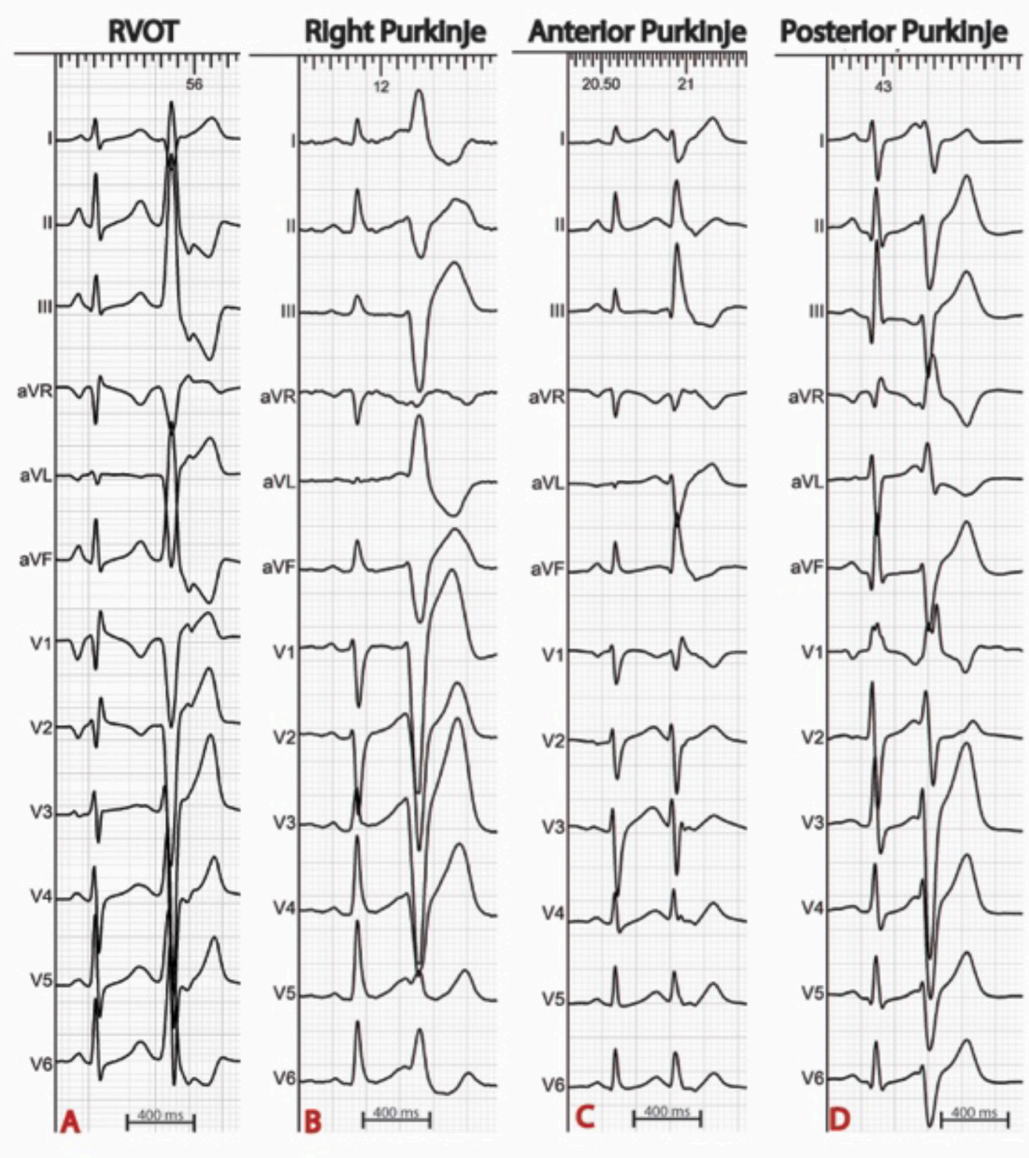
Examples of ECGs in patients with IVF. The origin of premature ventricular complexes triggering VF may be the right ventricular outflow tract **(A)**, the right Purkinje system **(B)**, or the left anterior **(C)** or posterior **(D)** Purkinje system.

The procedure is best scheduled during or as soon as possible after an electrical storm, a period during which the culprit PVCs tend to be frequent. Thereafter, PVCs become less frequent which reduces the likelihood of success. The PVC morphology on the 12 lead ECG is of particular interest as it guides mapping techniques and allows focus on the area of interest (Figure 1). When originating from the left Purkinje fibers, the PVCs are usually narrow (<120 ms) with a right-bundle-branch block morphology (6, 7, 11). They demonstrate right or left axis deviation when originating from the anterior or the posterior Purkinje fibers respectively. Discrete morphology changes are frequently observed in the left Purkinje PVCs. When originating from the right Purkinje arborization, the PVCs are usually wide and have a left-bundle-branch block morphology (6, 7, 11). More rarely, PVCs can originate from a non-conductive tissue source, particularly from the RVOT. In this case, the PVCs have an inferior axis. The coupling interval is classically variable and VF is usually triggered by short coupled PVCs. Discrete PVC morphology changes are frequently observed before VF initiation, potentially due to different exit sites.

As described by our group (6, 7, 16), the PVC origin may be mapped endocardially and is located at the earliest electrogram site relative to the onset of the PVC on the 12 lead ECG. The right ventricle is accessed by a venous femoral approach using a long sheath. The left ventricle is accessed either by a transeptal approach or a by a retrograde approach. The transeptal approach is effective in reaching most of the left ventricular myocardium and may provide more stability to map and ablate the anterior Purkinje and the antero-lateral papillary muscle. The retrograde approach is more effective in accessing the left basal septum and the left ventricular outflow tract. The transeptal approach is preferred in patients with aortic atherosclerosis and in the presence of aortic valve stenosis. A decapolar catheter is helpful in mapping the His-Purkinje arborization in both chambers. A lasso catheter can be used to map the RVOT (68). A multispline catheter is also useful for mapping over a wide area of ventricular endocardium with high spatial sampling and resolution.

**Figure 2:**
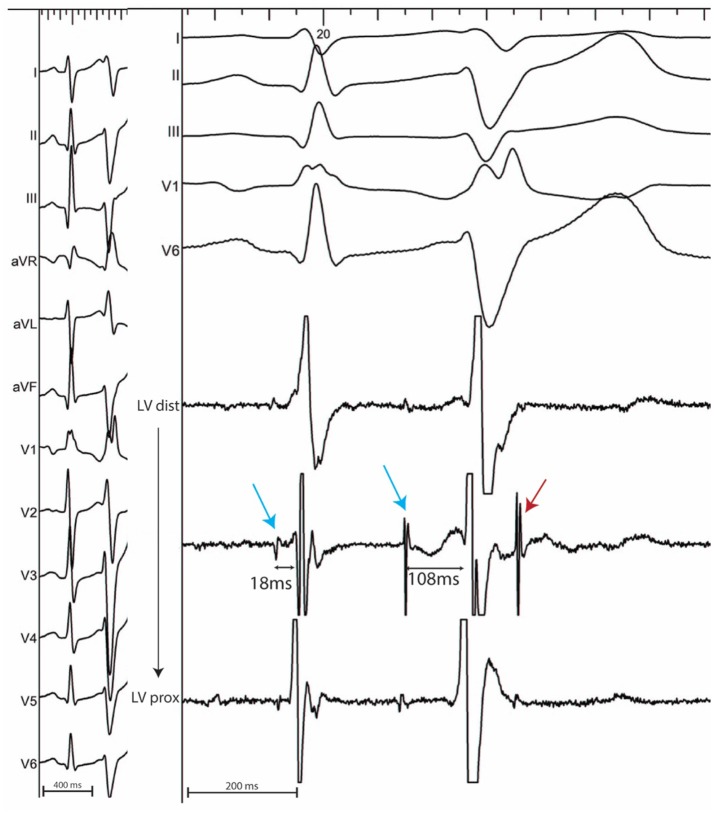
12 lead ECG with associated endocardial electrograms of a PVC arising from the posterior Purkinje network. Purkinje fascicular potentials precede QRS onset by 18 ms during sinus rhythm. During the PVC, Purkinje potentials precede QRS onset by 108 ms (blue arrows). Notice the presence of a concealed Purkinje potential (red arrow).

**Figure 3:**
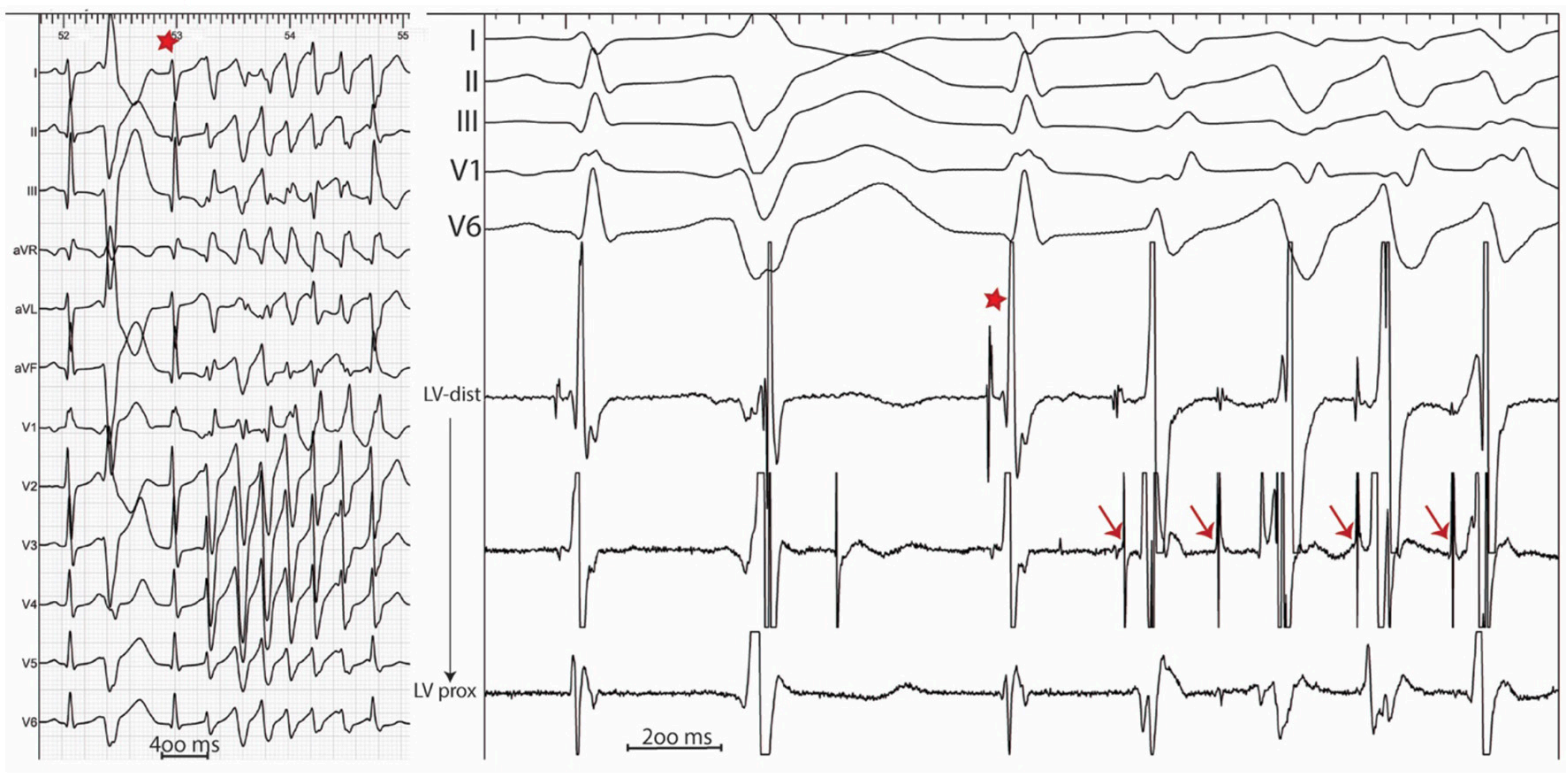
12 lead ECG **(Left)** with associated endocardial tracings **(Right)** showing spontaneous polymorphic PVCs from a patient with idiopathic VF. A wide PVC likely originating from the right ventricle is followed by a concealed retrograde Purkinje potential (red star). Purkinje potentials during sinus rhythm are shown by blue arrows. PVCs originating from the Purkinje fibers are preceded by Purkinje potentials with a different coupling interval (red arrows). Notice the modifications in PVC morphology which result from the complex arborization of the left Purkinje system.

In sinus rhythm, in the absence of intraventricular conduction abnormalities, distal Purkinje potentials are usually sharp (≤10 ms) and precede the QRS complex by ≤15 ms. Longer intervals indicate a fascicular origin. During a PVC, Purkinje potentials precede the local EGM by variable intervals that are usually greater than 15 ms (Figure 2). Purkinje activation can be blocked and concealed and can be activated retrogradely (Figure 3). Purkinje potentials become concealed within the local EGMs in the presence of intraventricular conduction abnormalities. Therefore, special care should be made during mapping to avoid inadvertent bumping of the left or right bundles. The absence of Purkinje potential at the earliest ventricular activation site during sinus rhythm indicates a myocardial origin. Whenever needed, PVCs can be induced by pacing maneuvers (atrial or ventricular) and/or more rarely by intravenous infusion of Isoproterenol (1–2 mcg/kg/min) or Ajmaline (1 mg/kg).

In the absence of spontaneous or inducible PVCs, pace-mapping may give an indication of the area of interest. However, pace-mapping cannot reliably reproduce the morphology of the Purkinje triggered PVCs due to simultaneous capture of the surrounding myocardium. Pace mapping is performed with the lowest pacing output (twice the diastolic threshold, range 2–15 mA) with a pulse width of 2 ms in order to capture the local ventricular myocardium. Different systems allow analysis of the degree of similarity between the recorded PVC and the original one and express it as a percentage. This comparison may also be performed for mechanically induced PVCs.

**Figure 4:**
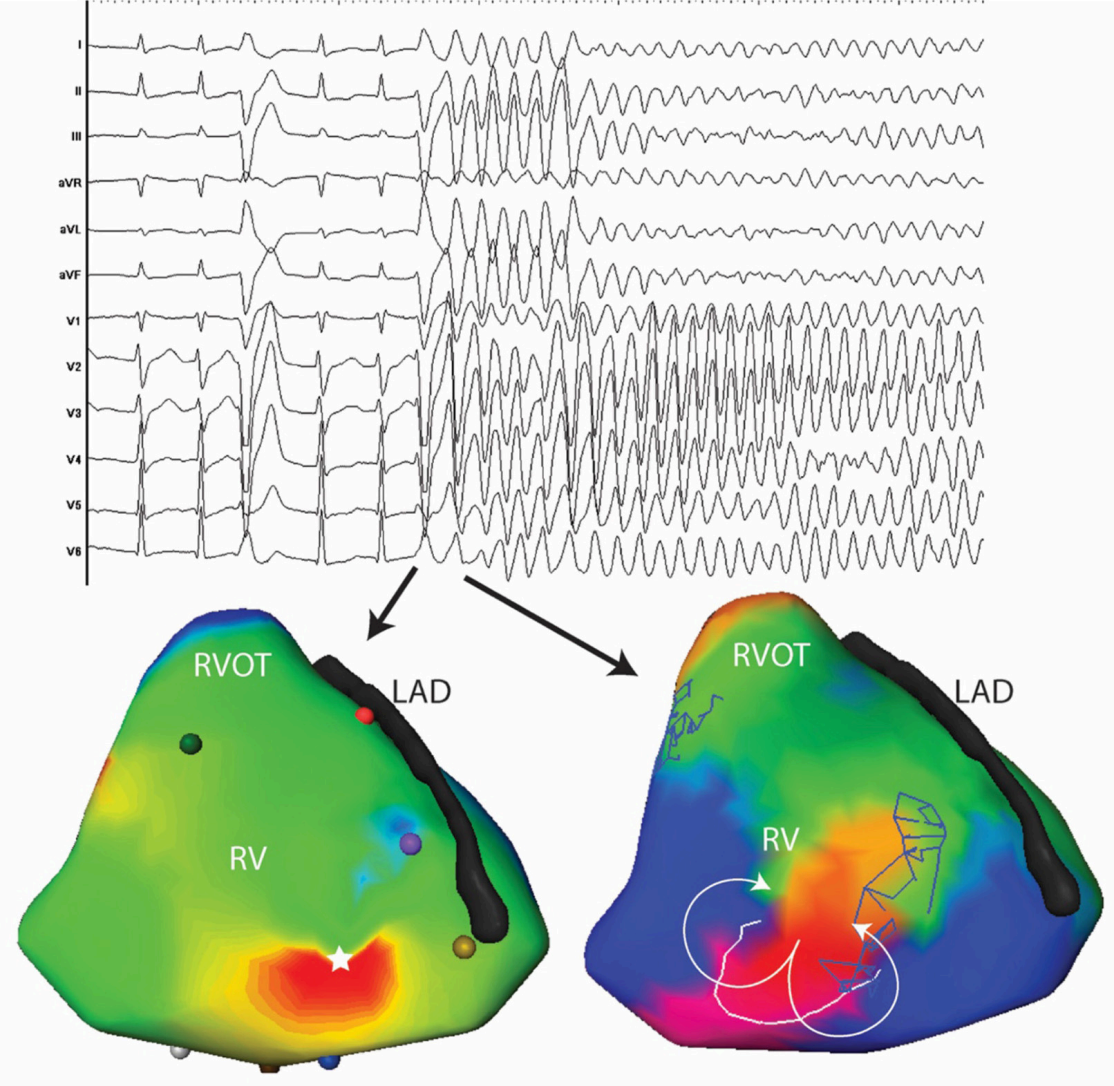
Twelve lead ECG and activation maps of the first and second beats of spontaneously initiated VF in a 30-year-old man. The PVC initiating VF has a similar morphology as the previous PVC with subtle changes in the precordial leads (V2-V3). The PVC initiating VF is located at the antero-apical RV (white star). The subsequent beat is a figure of eight at the same site as the first PVC.

Electrocardiographic imaging (ECGi) represents an additional tool that may accurately identify the origin of the PVCs triggering VF (69–71). It is of particular interest in patients with rare PVCs (Figure 4).

## Is there a substrate in patients with IVF?

In order to sustain, VF requires a substrate, either anatomical or electrical. However, current diagnostic tools are limited and may miss subtle structural abnormalities. In addition, the lack of mapping resolution during VF in humans, as well as the unknown effects of acute or dynamic phenomena, may explain the lack of data in this group.

In a recent study (20), we evaluated 24 patients who survived IVF. All patients benefited from non-invasive mapping to characterize the drivers maintaining VF during the initial 5 s of VF. In addition, all patients benefited from high density endocardial and epicardial biventricular mapping. A decapolar catheter was used to map the endocardium of the right and left ventricles, while 20-pole catheters with 2 mm inter-electrode spacing (Pentaray, BiosenseWebster, CA; Lasso, BiosenseWebster, CA) were used for biventricular epicardial mapping.

**Figure 5:**
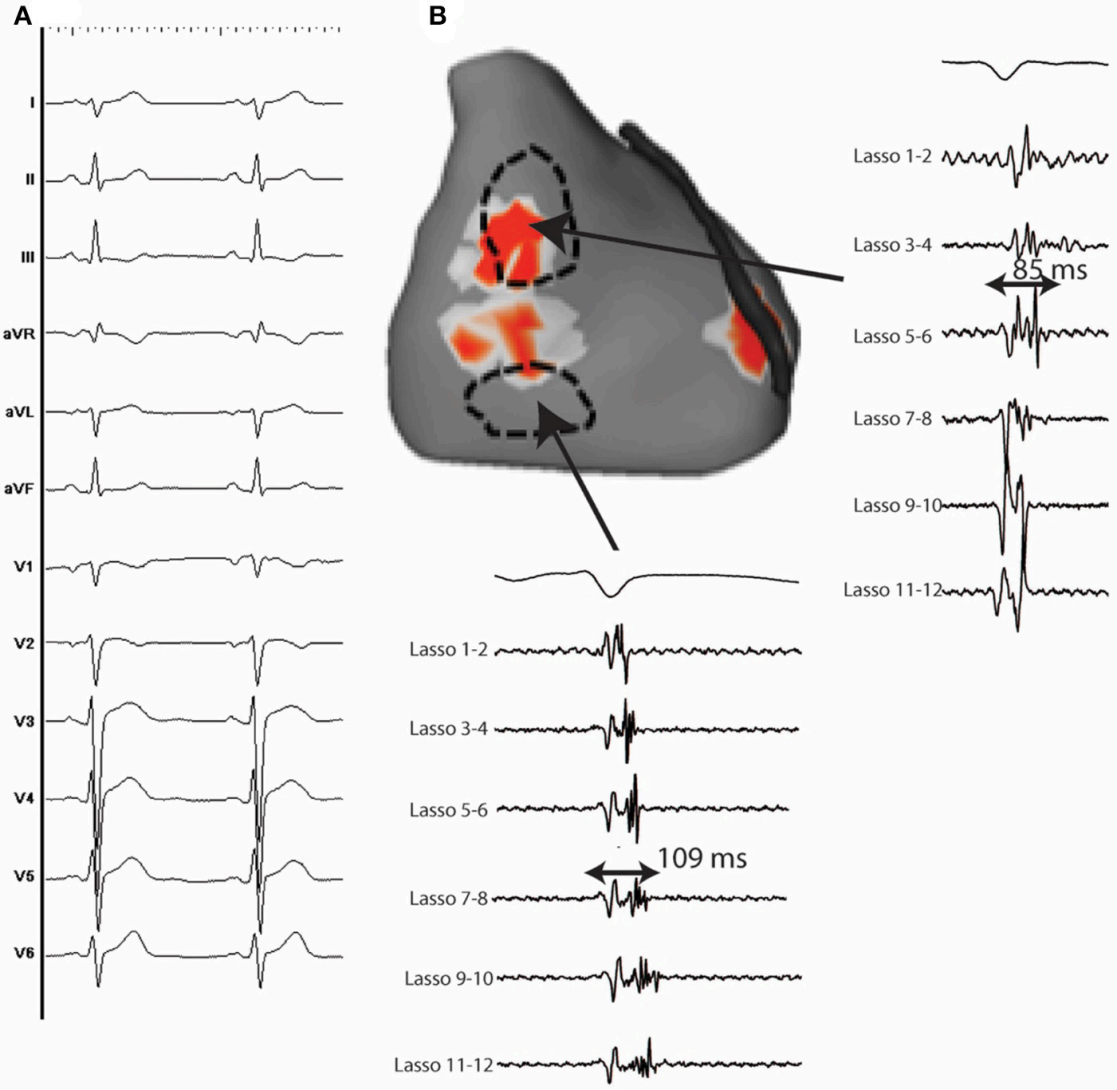
**(A)** 12 lead ECG of a 37 y.o man with IVF. **(B)** Anterior view of the heart showing an area of reentrant activity located at the anterior and lateral epicardial RV. Fragmented epicardial electrograms with long duration during sinus rhythm are identified close to the driver sites.

A total of 19 VF episodes were analyzed. VF occurred spontaneously in 3 patients and was induced by electrical stimulation in 16, whereas it was not inducible in 5 patients. A mean of 28 ± 3 VF cycles were recorded during the initial 5 s and the mean VF cycle length was 183 ± 23 ms. A mean of 2.8 ± 0.7 activities (including focal and reentrant activities) were recorded per cycle, being reentrant in 87% and focal in 13%. A ventricle was considered as dominant when hosting more than 50% of the activities during the initial 5 s. This was the case in 9 patients while the rest demonstrated biventricular distribution of the fibrillatory activities. High density mapping during sinus rhythm identified abnormal electrograms [>70 ms duration and more than 3 spikes (72–74)] in 15/24 patients (62.5%) (Figure 5). They were arranged in a confluent (rather than a distributed) pattern and covered a limited surface area (13 ± 6 cm^2^), representing 5 ± 3% of the total ventricular surface area. The abnormal electrograms were located in the right ventricle in 11, the left ventricle in one and both in three, and were predominantly epicardial. The localized substrate colocated with the driver regions in 76% of cases (*p* < 0.001). The 9 patients without structural alterations had a high incidence of Purkinje triggers (7/9).

## Ablation strategies and procedural outcome

The site of earliest ventricular activation during spontaneous PVCs is the target of choice. In patients without clinical PVCs, ablation can target the local Purkinje potentials or the site of best matched morphology by pace-mapping. Ablation may be performed using an irrigated 3.5 mm tip catheter. Power is delivered according to catheter location. PVCs originating from the RVOT or from the Purkinje network are ablated using 30 watts. The power can be increased to 50 watts on the septum when the PVC origin is intramural. Manual titration of the irrigation flow is performed to achieve the required power. In all cases, ablation is extended approximately 1–2 cm^2^ around the target site. During ablation, it is common to have exacerbation of the arrhythmia (multiple PVCs leading to polymorphic VT and more rarely to VF) before the eradication of premature beats. The occurrence of QRS widening during ablation indicates potential catheter displacement toward the more proximal conduction system and ablation should be stopped (7). Knecht et al. (11) have reported the occurrence of transient left bundle branch block in one patient and nonspecific intraventricular conduction defects in 6 of 38 patients. The procedural endpoint is complete elimination of the culprit PVC and of the local Purkinje potentials. Acute procedural success rate is high. In their initial report, Haissaguerre et al. (7) achieved complete elimination of all the clinical PVCs that were recorded in 24 of 27 patients. Ablation was guided by pace-mapping in the remaining 3 patients. Two patients had early recurrence of PVCs with different morphologies that were successfully eliminated during a second procedure. Knecht et al. reported successful elimination of the culprit PVCs in all patients who presented with spontaneous PVCs.

The localized substrate identified during mapping represents a novel additional target for ablation. In a recent study, we targeted the abnormal substrate in 12 patients with IVF with recurrent episodes. Ablation was associated with VF free outcome with 14-months follow-up (20).

**Figure 6:**
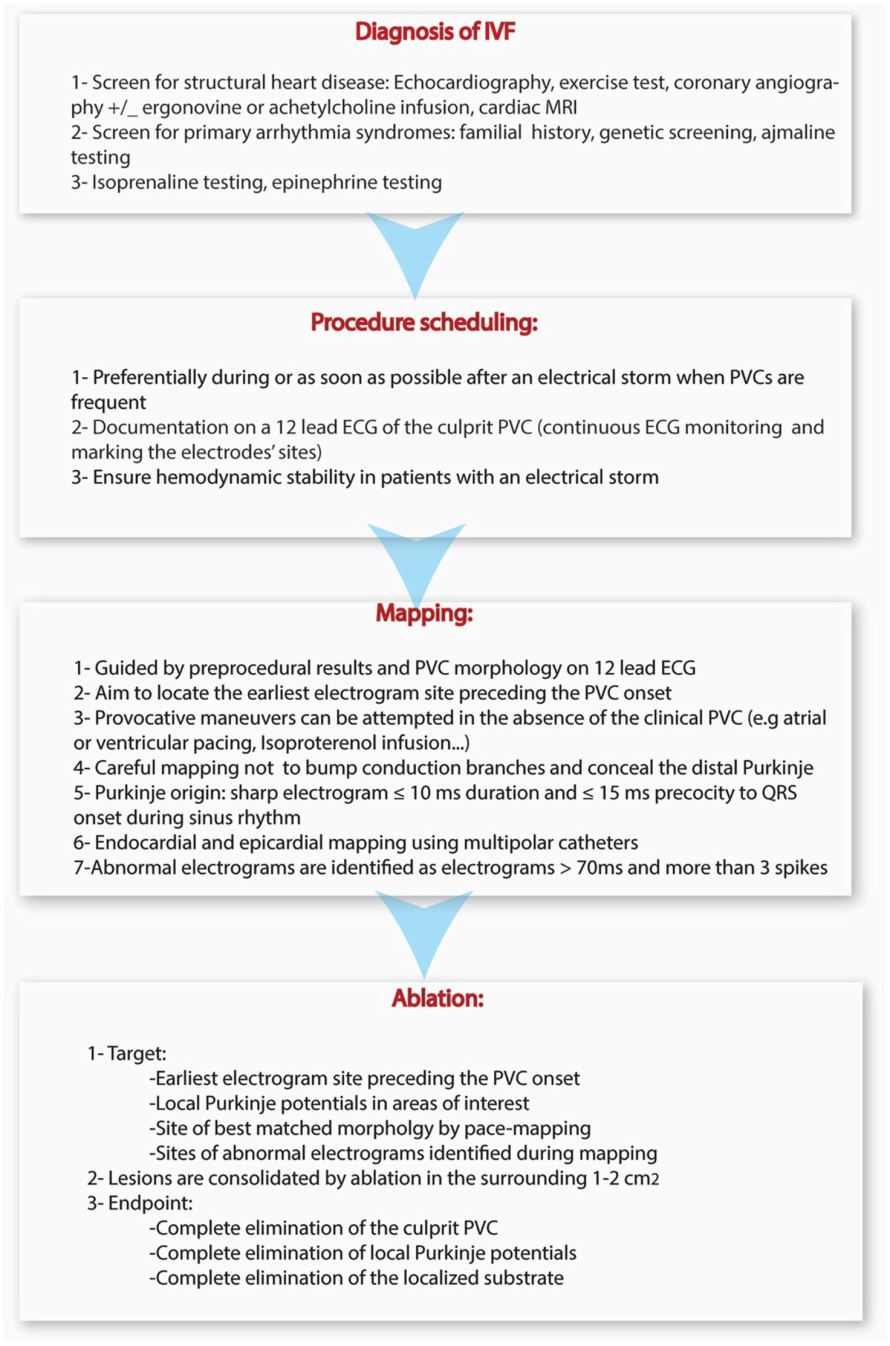
Summary of the current diagnostic, mapping and ablation approaches in patients with IVF.

A summary of the diagnostic, mapping and ablation approaches in patients with IVF is provided in Figure 6.

## Procedural outcomes

Clinical and Holter monitoring is performed in all patients for at least 3 days after the procedure. ICDs are systematically implanted before discharge if not already *in situ*. Antiarrhythmic drugs are continued for at least 3 months after the procedure. After discharge, patients are followed-up at 1, 3, 6, and 12 months, then every 6 months to 1 year thereafter. Follow-up includes clinical examination, 12-lead ECG, exercise test and ICD interrogation.

Following current guidelines (3, 4), we systematically perform familial screening, including resting ECG, exercise testing and echocardiography in first degree relatives. In selected cases, Holter and signal-averaged ECGs, MRI and provocation testing (including with Class Ic antiarrhythmic drugs and epinephrine) are performed.

To date, the greatest experience with mapping and ablation of IVF was reported in a multicenter trial of 38 patients (11). After a mean follow-up of 63 months, 31 of 38 (82%) patients were free from VF recurrence. VF recurrence occurred in the remaining 7 (18%) patients after a median of 4 months with multiple episodes in 5 of them. The presence of bundle branch block before ablation was the only parameter associated with worse outcome and with VF recurrence (11). There was no difference in outcome between patients with Purkinje triggers and those with muscular triggers.

## Conclusions

Idiopathic VF is diagnosed in around one third of survivors of unexplained SCD aged under 35 years. Genetic testing allows identification of a likely causative mutation in around one quarter of unexplained sudden deaths in children and young adults. Ablation of the PVCs that trigger VF in this setting is associated with high rates of acute success and long-term freedom from VF recurrence. Importantly, almost two thirds of patients have subtle structural abnormalities identified by high density electrogram mapping and missed by current imaging tools. This localized substrate, which colocates with regions of VF drivers, provides an explanation for so called unexplained SCD and represents a novel potential target for ablation.

## Author contributions

All authors listed have made a substantial, direct and intellectual contribution to the work, and approved it for publication.

### Conflict of interest statement

The authors declare that the research was conducted in the absence of any commercial or financial relationships that could be construed as a potential conflict of interest.
